# Newborn screening for primary carnitine deficiency: who will benefit? – a retrospective cohort study

**DOI:** 10.1136/jmg-2023-109206

**Published:** 2023-07-24

**Authors:** Loek Crefcoeur, Sacha Ferdinandusse, Saskia N van der Crabben, Eugènie Dekkers, Sabine A Fuchs, Hidde Huidekoper, Mirian Janssen, Janneke Langendonk, Rose Maase, Monique de Sain, Estela Rubio, Francjan J van Spronsen, Frédéric Maxime Vaz, Rendelien Verschoof, Maaike de Vries, Frits Wijburg, Gepke Visser, Mirjam Langeveld

**Affiliations:** 1 Metabolic Diseases, Wilhelmina Children's Hospital, University Medical Centre, Utrecht, The Netherlands; 2 Department of Pediatrics, Division of Metabolic Disorders, Amsterdam UMC Locatie AMC, Amsterdam, The Netherlands; 3 Clinical Chemistry, Laboratory Genetic Metabolic Diseases, Amsterdam UMC Locatie Meibergdreef, Amsterdam, The Netherlands; 4 Human Genetics, Amsterdam UMC Locatie Meibergdreef, Amsterdam, The Netherlands; 5 United for Metabolic Diseases, Amsterdam, The Netherlands; 6 Centre for Population Screening, RIVM, Bilthoven, The Netherlands; 7 Department of Pediatrics, Center for Lysosomal and Metabolic Diseases, Erasmus Medical Center, Rotterdam, The Netherlands; 8 Department of Internal Medicine, Radboud University Medical Center, Nijmegen, The Netherlands; 9 Department of Internal Medicine, Center for Lysosomal and Metabolic Diseases, Erasmus Medical Center, Rotterdam, The Netherlands; 10 Department of Biologicals, Screening and Innovation, RIVM, Bilthoven, The Netherlands; 11 Section Metabolic Diagnostics, Department of Genetics, Wilhelmina Children's Hospital, University Medical Centre, Utrecht, The Netherlands; 12 Department of Pediatrics/Laboratory of Clinical Genetics, Maastricht UMC+, Maastricht, The Netherlands; 13 Section of Metabolic Diseases, University Medical Centre Groningen, Beatrix Children's Hospital, Groningen, The Netherlands; 14 Laboratory Genetic Metabolic Diseases, Amsterdam UMC, University of Amsterdam, Departments of Clinical Chemistry and Pediatrics, Core Facility Metabolomics, Emma Children’s Hospital, Amsterdam Gastroenterology Endocrinology Metabolism, Amsterdam UMC Locatie Meibergdreef, Amsterdam, The Netherlands; 15 Department for Vaccine Supply and Prevention Programs, RIVM, Bilthoven, The Netherlands; 16 Department of Pediatrics, Amalia Children's Hospital, Radboud University Medical Center, Nijmegen, The Netherlands; 17 Department of Endocrinology and Metabolism, Amsterdam UMC Locatie Meibergdreef, Amsterdam, The Netherlands

**Keywords:** Neonatal Screening, Pathological Conditions, Signs and Symptoms, Pediatrics, Human Genetics, Public Health

## Abstract

**Background:**

Newborn screening (NBS) programmes identify a wide range of disease phenotypes, which raises the question whether early identification and treatment is beneficial for all. This study aims to answer this question for primary carnitine deficiency (PCD) taking into account that NBS for PCD identifies newborns with PCD and also until then undiagnosed mothers.

**Methods:**

We investigated clinical, genetic (variants in *SLC22A5* gene) and functional (carnitine transport activity in fibroblasts) characteristics of all referred individuals through NBS (newborns and mothers) and clinically diagnosed patients with PCD (not through NBS). Disease phenotype in newborns was predicted using data from PCD mothers and cases published in literature with identical *SLC22A5* variants.

**Results:**

PCD was confirmed in 19/131 referred newborns, 37/82 referred mothers and 5 clinically diagnosed patients. Severe symptoms were observed in all clinically diagnosed patients, 1 newborn and none of the mothers identified by NBS. PCD was classified as severe in all 5 clinically diagnosed patients, 3/19 newborns and 1/37 mothers; as benign in 8/19 newborns and 36/37 mothers and as unknown in 8/19 newborns. Carnitine transport activity completely separated severe phenotype from benign phenotype (median (range): 4.0% (3.5–5.0)] vs 26% (9.5–42.5), respectively).

**Conclusion:**

The majority of mothers and a significant proportion of newborns with PCD identified through NBS are likely to remain asymptomatic without early treatment. Conversely, a small proportion of newborns with predicted severe PCD could greatly benefit from early treatment. Genetic variants and carnitine transport activity can be used to distinguish between these groups.

**WHAT IS ALREADY KNOWN ON THIS TOPIC**

- Primary carnitine deficiency (PCD) is an inborn error of metabolism that may cause severe symptoms early in life, but some affected individuals can remain asymptomatic throughout life without treatment.

**WHAT THIS STUDY ADDS**

- The majority of individuals with PCD identified by newborn screening (NBS) (in this study 78%) are unlikely to develop severe symptoms early in life. For a minority of newborns with PCD (in this study at least 16%), early treatment is likely to prevent severe disease. There were significant differences between these groups with respect to carnitine transport activity in fibroblasts (4% in the severe vs 27% in the mild/asymptomatic group) and the genetic variants identified.

**HOW THIS STUDY MIGHT AFFECT RESEARCH, PRACTICE OR POLICY**

- The residual carnitine transport activity, in conjunction with genetic analysis, can be used to distinguish severe PCD from a benign form of the disorder, aiding clinical decision making after NBS.

## Introduction

Newborn screening (NBS) programmes enable an accelerated diagnosis of severe conditions, which allows early treatment initiation and prevention of potentially irreversible signs and symptoms. Expansion of NBS programmes often results in the detection of newborns with previously unreported genotypes, not found in clinically diagnosed patients before inclusion of the disease in NBS panels.^1–3^ As a result, there are little or no data allowing prediction of the disease course in individuals with clinically unreported genotypes.^4 5^ Nevertheless, treatment is generally initiated in all diagnosed newborns, which complicates evaluation of the natural, untreated, disease course in this group.

Primary carnitine deficiency (PCD) (OMIM #212140) is an autosomal-recessive disorder caused by variants in the *SLC22A5* gene, which encodes the Organic Cation Transporter Novel 2 (OCTN2) protein.^6^ OCTN2 maintains the intracellular carnitine concentrations through transport of carnitine into cells, including reabsorption of carnitine in the renal tubuli.^7 8^ Impaired function of OCTN2 leads to decreased intracellular free carnitine concentrations. In case of carnitine deficiency, activated long-chain fatty acids are not efficiently transported into the mitochondrial matrix for oxidation and ketone body production. Clinically identified patients present, mainly in childhood (90%) with myopathy, (hepatic) encephalopathy, cardiomyopathy and/or arrhythmia and this condition is potentially fatal.^9^ Sudden cardiac death has been observed in adulthood.^9^ Treatment is simple and consists of lifelong oral supplementation of carnitine. The potential negative effect of treatment can be a fishy body odour.

Because of the severity of the symptoms and the availability of an effective treatment, PCD has been included in NBS panels in a number of countries.^10 11^ Screening is conducted by the measurement of the free carnitine concentrations in dried blood spots (DBS). Unexpectedly, inclusion of PCD in NBS panels led to the detection of mothers with PCD, diagnosed when their child (unaffected by PCD) was referred because of low free carnitine concentration in the NBS DBS, which was caused by a low free carnitine concentration in the mother.^10 12 13^ Most of these mothers were asymptomatic at the time of diagnosis and without previous symptoms that might be related to PCD.^9 14^ Nevertheless, since cardiac events have been reported in a small number of previously asymptomatic adult patients with PCD, these newly diagnosed mothers were treated and followed up in regular care.^9 15 16^ However, it may well be that a large proportion of these mothers with low carnitine transporter activity will never become symptomatic without treatment. The high number of patients identified by NBS with previously unreported genotypes hampers prognostication. In addition, the detection of mothers with mild disease or no disease at all raises doubts regarding the benefit of inclusion of PCD in NBS programmes. The incomplete understanding of benefit of screening, weighed against potential harm caused by identification and treatment of asymptomatic individuals, led to discontinuation of NBS for PCD in New Zealand.^17^

In the Dutch NBS programme, free carnitine levels are monitored in all DBS as a control for the quality of the acylcarnitine measurements. Low carnitine in DBS may cause unreliable screening results for a number of disorders relying on the acylcarnitine profile, including long-chain and medium-chain fatty acid oxidation disorders.^18 19^ Individuals with low carnitine levels are therefore referred, which might lead to the diagnosis PCD, although PCD is not officially included in the Dutch NBS panel. Now, after more than a decade of detecting PCD as an incidental finding of NBS in the Netherlands, we can evaluate the yield of referrals because of low free carnitine in NBS DBS’s. The detection of maternal PCD cases provides a unique opportunity to assess the natural course of PCD detected by screening. The presented study aims to evaluate patient characteristics that can be used to identify those patients that benefit from early treatment and those that do not require medical care and can be regarded as healthy individuals with a benign metabolic trait.

## Methods

### Study design

Written informed consent was obtained from all participants and/or their caregivers prior to enrolment. The study was conducted in accordance with the principles of the Declaration of Helsinki.

### Participants and data collection

The first group eligible for inclusion were all patients in the Netherlands diagnosed with PCD (further referred to as clinically diagnosed patients). PCD diagnosis was based on a genetic confirmation of two variants in *SLC22A5*, and/or a functional confirmation with a reduced residual carnitine transport activity, defined as <50% transport activity of controls in cultured skin fibroblasts. The second eligible group consisted of all newborns referred because of low carnitine concentration in NBS (further referred to as newborns). The third group encompassed all mothers referred because of unexplained low carnitine in the DBS of their child (further referred to as mothers). Inclusion ended on 31 December 2020. The following data were collected retrospectively: medical history, perinatal data, family history, physical examinations, education and occupation, diet, blood/serum/urine free carnitine concentrations, DNA diagnostics and additional test results performed in the context of evaluation of PCD (eg, ECG or cardiac ultrasound). In addition, those diagnosed with PCD were either seen or contacted by phone by the researcher for an additional questionnaire on their current health status.

### Newborn screening in the Netherlands

During the inclusion period (2007–2020) 2 464 710 children were screened (99.4% of total births) and 98.5% of NBS-samples were obtained within 72–168 hours after birth (the target range for NBS-sampling in the Netherlands). The acylcarnitine profile was determined in DBS. In case of a low free carnitine concentration (≤4 µmol/L in the period of January 2007 until June 2009 and ≤ 5 µmol/L from July 2009 onwards), a repeat sample was taken within 10 days of the initial detection of low free carnitine in the first dried blood spot. When low free carnitine concentration was also present in the second sample (applying the same cut-off value), the newborn was referred to a regional metabolic centre for follow-up, preferably within 24 hours. As a result of the evaluation at the metabolic centre, the mother of the newborn could then be referred to an adult metabolic specialist. The referral rate of mothers increased over time, as more reports of maternal PCD cases identified through NBS were published.^10 13 14^

### Carnitine transport assay

Residual carnitine transport activity was analysed in cultured skin fibroblasts according to the method described by Ferdinandusse *et al* (novel assay).^20^ All cell lines were analysed in two independent experiments and the measurements within the experiments were performed in duplicate. Reported values are the mean of all measurements and are expressed as percentage of the mean activity in two or three control cell lines analysed in parallel.

### Genetic data

Analysis of the *SLC22A5* gene was performed by sequence analysis of all exons and flanking intronic sequences amplified by PCR from genomic DNA isolated from either fibroblasts or blood from patients. The reference sequence used was RefSeq NM_003060.3. Variants were annotated using Alamut Visual V2.11 (Interactive Biosoftware). All variants with classification 3 or higher were recorded. These variants were subsequently grouped into (1) missense variants and (2) nonsense, frameshift and splice-site variants.

We annotated the identified *SLC22A5* variants as either *classic variants*: variants previously identified in patients diagnosed following presentation with disease symptoms (either in our cohort or published in literature) or as *screening variants*: variants identified in patients following NBS screening diagnosis only (current cohort and published literature) (online supplemental table 1).

10.1136/jmg-2023-109206.supp1 Supplementary data

### Disease severity classification

We developed a disease severity classification for all genotypes found in patients in the current study, using clinical information from our own study population, as well as available data from published cases with the exact same combination of variants identified in the *SLC22A5* gene. By combining all available clinical data from adult untreated cases with the same genotypes, this classification aims to correct for the bias created by early initiation of treatment in NBS children, causing a benign disease course. Three PCD disease severity categories were defined: (1) *Severe*: patients with severe disease are those who suffered severe symptoms (this cohort) and patients from this study cohort with genotypes previously reported in patients that suffered severe symptoms; (2) *Likely benign*: individuals with a likely benign metabolic trait are adult patients in this study cohort who did not suffer any events or severe disease symptoms and newborns in this cohort with genotypes reported in literature only in untreated patients that remained asymptomatic or had mild symptoms into adulthood and (3) *Unknown*: Individuals for whom the significance of their metabolic trait is unknown are treated newborns (this cohort) with genotypes never reported in untreated patients in literature.

For this classification, severe symptoms were considered those that may cause irreversible damage: sudden death, cardiac arrest, ventricular fibrillation, sustained ventricular tachycardia (ventricular rhythm, with a QRS-complex >120 ms) that is faster than 100 bpm, that lasts at least 30 s), cardiomyopathy with cardiac failure (left ventricular ejection fraction <50%), encephalopathy, coma (without a discernible external cause), hypoglycaemia (blood glucose <2.5 mmol/L), sepsis-like presentation and rhabdomyolysis (plasma Creatine Kinase (CK) >5 times the upper reference range). Fatigue and myalgia reported in the medical history of patients (without elevated plasma CK) were annotated as mild symptoms. Because of the high risk of observational selection bias, new complaints of fatigue and/or myalgia that were mentioned only after specific enquiry by the physician/researcher after PCD was suspected or diagnosed were recorded, but not considered a symptomatic involvement in the context of this study.

### Statistics

Statistical analyses were performed using R (V.3.6.2, R Core Team, Vienna, Austria). Continuous data were tested for normality with Shapiro-Wilk’s method. The two-sided T-test was used to compare normally distributed continuous data between two groups and the Mann-Whitney U test was used to compare non-normally distributed continuous data between two groups. The χ^2^ test was used for comparison of categorical data. Multiple comparisons were adjusted with the Bonferroni correction. Significance was assumed for p<0.05.

## Results

**Figure 1:**
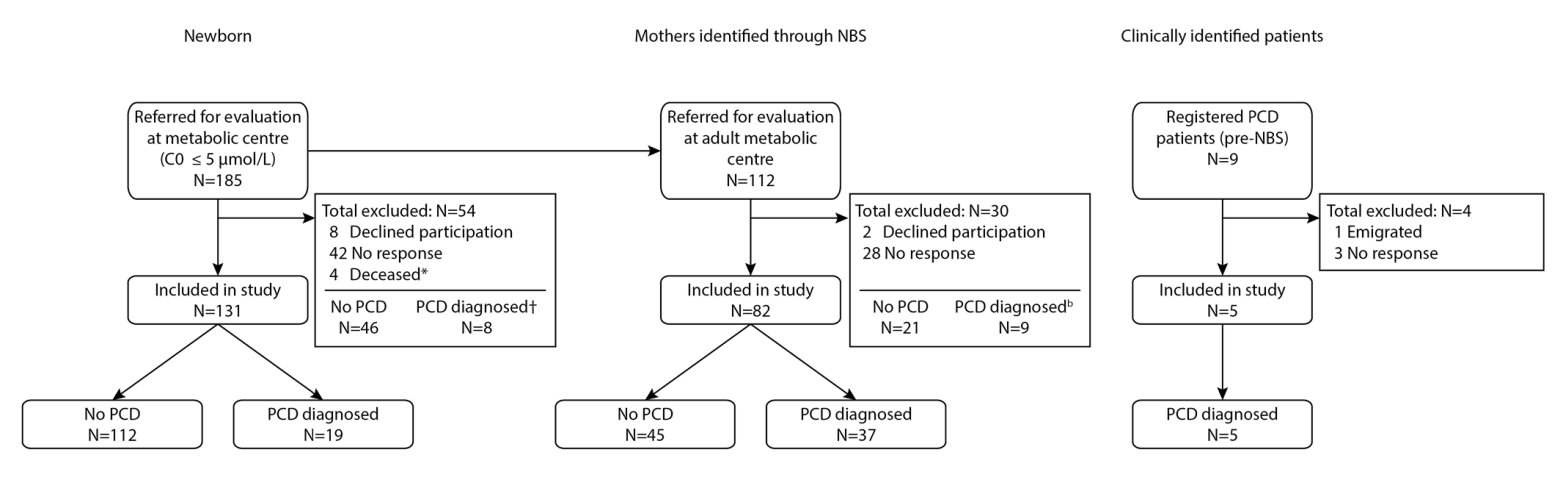
Flowchart of patient inclusion. *All four infants died from complications explained by extremely preterm birth. †Based on national registries, validity of diagnosis could not be confirmed. NBS, newborn screening; PCD, primary carnitine deficiency.

From 2007 until 2020, 185 newborns were referred because of low free carnitine concentrations detected by NBS and 112 mothers were referred secondary to their newborn (see figure 1). The parents of 131 newborns and 82 mothers consented to participation in the study. Five of in total nine known clinically diagnosed Dutch patients (all diagnosed before free carnitine was included in the Dutch NBS programme) were included in the study.

### Newborn screening results

An overview of clinical, biochemical and genetic characteristics of all patients with confirmed PCD is provided in table 1. Diagnosis of PCD was confirmed in 19/131 (14.5%) of the referred newborns and 37/82 (45.1%) of the referred mothers. No inherited metabolic disorders other than PCD were identified in newborns. Five mothers were diagnosed with another inherited metabolic disorder, causing the low carnitine concentration in the DBS of their child (medium-chain acyl-CoA dehydrogenase deficiency, n=2; glutaric aciduria type 1, n=2; 3-methylcrotonyl-CoA carboxylase deficiency, n=1). Characteristics of all referred individuals are provided in online supplemental table 2.

10.1136/jmg-2023-109206.supp2 Supplementary data

**Table 1:** Clinical, biochemical and functional characteristics of patients with PCD in the study cohort

	Newborns (n=19)		Mothers (n=37)		Clinically diagnosed (n=4)	
Sex (male)	9 (47.4%)	{0}	0 (0%)	{0}	2 (40.0%)	{0}
Gestational age at birth (weeks)	40.1 (37.3, 41.7)	{1}	–		–	
Birth weight (g)	3390 (2490, 4300)	{0}	–		–	
Sibling death	0 (0%)	{5}	1 (3.2%)	{6}	1 (20.0%)	{0}
Age at diagnosis (years)	0 (0, 0)	{0}	31.0 (21.0, 40.0)	{6}	1.0 (1.0, 15.0)	{0}
Age at last follow-up (years)	4.6 (1.0, 14.1)	{0}	34.6 (24.8, 48.6)	{0}	20.7 (19.6, 37.4)	{0}
Symptoms		{0}		{0}		{0}
Asymptomatic	15 (78.9%)		29 (78.4%)		0 (0%)	
Self-reported signs*	3 (15.8%)		8 (21.6%)		0 (0%)	
Severe symptoms†	1 (5.3%)		0 (0%)		5 (100%)	
*SLC22A5* variants		{0}		{0}		{0}
Missense	13 (68.3%)		33 (89.2%)		3 (60.0%)	
Missense/Null	4 (21.1%)		2 (5.4%)		0 (0%)	
Null	2 (10.5%)		0 (0%)		2 (40.0%)	
Missense, single variant identified	0 (0%)		2 (5.4%) *		0 (0%)	
Variant reported		{0}		{0}		{0}
2 classic variants	5 (26.3%)		1 (2.7%)		5 (100%)	
2 screening variants	7 (36.8%)		31 (83.7%)		0 (0%)	
1 classic and 1 screening variant	7 (36.8%)		3 (8.1%)		0 (0%)	
Single variant identified (screening variant)§	0 (0%)		2 (5.4%)*		0 (0%)	
Transport activity in fibroblasts (% of controls)	11.0 (3.5, 27.0)	{14}	26.0 (4.0, 42.5)	{17}	4.3(4.0, 5.0)	{1}
Free carnitine concentration (blood; μmol/L)						
First NBS DBS sample‡	3.5 (1.8, 4.8)	{0}	3.7 (1.9, 5.0)	{2}		
Second NBS DBS sample‡	3.1 (1.3, 4.7)	{1}	3.8 (2.0, 5.0)	{2}		
First sample at metabolic centre (plasma)	7.5 (2.0, 12.0)	{0}	6.6 (2.1, 18.8)	{3}	3.00(1.0, 4.4)	{0}

Data presented as n (%) or median (Min–Max). Missing data points are presented in grey, within braces.

*Fatigue, myalgia or corrected motor developmental delay.

†Sepsis-like-presentation, cardiomyopathy, arrhythmia and/or coma.

‡For mothers: concentration in the NBS sample of their child.

§Diagnosis based on reduced transport activity (30.5% and 40.5%, respectively).

DBS, dried blood spot; IEM, inborn error of metabolism; NBS, newborn screening; PCD, primary carnitine deficiency.

### Clinical characteristics of patients with confirmed PCD

The median age at last follow-up of the 19 newborns diagnosed with PCD was 4.6 years. Fifteen (79%) were asymptomatic, three (21%) had self-reported signs (fatigue n=2; myalgia n=1) and one patient presented with a sepsis-like episode with negative microbial cultures at 2 weeks of age. As she presented at the emergency room, her NBS results came in, after which she immediately received carnitine supplementation, and she quickly recovered. Of the mothers diagnosed with PCD (n=37), median age at diagnosis was 31 years, median age at last follow-up was 34.6 years and the total observed life years of mothers within the cohort was 1279 years. Twenty-nine mothers (78%) were asymptomatic, eight (22%) had self-reported symptoms prior to the diagnosis (fatigue n=6; myalgia n=1; non-specific pain in legs n=1) and none had experienced a severe event.

Clinically diagnosed patients (n=5) all presented in childhood (range 3 months to 15 years) with severe events (table 2, patients 8, 10, 11, 12 and 193). The median age at diagnosis was 1 year and median age at last follow-up was 20.7 years. Four patients presented with cardiomyopathy that completely resolved with carnitine supplementation, of whom two have been previously reported in literature (patients 10 and 193).^21 22^ One patient presented with ventricular fibrillation at 15 years of age. After defibrillation, episodes of non-sustained ventricular tachycardia persisted, and a cardiac defibrillator was implanted. Carnitine supplementation resulted in complete normalisation of cardiac parameters within 1 year.^23^ She moved abroad and was lost to follow-up 3 years later.

**Table 2:** Overview of patients with severe PCD

Patient ID	Identified	Age initial symptoms (Years)	Age at last follow-up (Years)	Initial symptoms	Clinical course at follow-up	Treatment effect	Variants DNA (Protein)	Transport activity
8	Clinically	<1	11–20	Sepsis-like presentation, Coma, Cardiomyopathy	Difficulty feeding	Complete resolution.	**c.396G>A;396G>A** (**p.Trp132Ter;Trp132Ter**)	NA
10	Clinically	<1	31–40	Cardiomyopathy	Cardiomyopathy at 30 years	Complete resolution within 1Y. After suspending treatment for 12Y cardiomyopathy recurred with severe cardiac failure.	c.632A>G;632A>G (p.Tyr211Cys;Tyr211Cys)	4%
11	Clinically	11–20	11–20	Ventricular fibrillation	NA	Defib. Implanted, normal ECG after 1Y.	c.1232G>T;1232G>T (p.Gly411Val; Gly411Val)	5%
12	Clinically	1–3	21–30	Cardiomyopathy	Fatigue	Complete resolution.	**c.844C>T;844C>T** (**p.Arg282Ter;Arg282Ter**)	4%
193	Clinically	<1	11–20	Cardiomyopathy, CMV infection, Failure to thrive	Hypoglycaemic coma with VF which lead to diagnosis at 1–3 years. Learning disability. Sudden death at 20 years	With conventional treatment (digoxine, diuretics) no improvement. On carnitine supplementation complete resolution.	c.632A>G;632A>G (p.Tyr211Cys;Tyr211Cys)	4.5%
502	NBS	<1	11–20	Sepsis-like presentation	Learning disability	Fast recovery after initiating carnitine supplementation.	**c.597delG;597delG** (**p.Phe200Leufs;Phe200Leufs**)	3,5%
131	Mother	31–40	31–40	None	Asymptomatic	None	c.248G>T;248G>T* (p.Arg83Leu;Arg83Leu)	4%
597†	NBS	NA	4–10	–	Asymptomatic	–	c.248G>T;248G>T* (p.Arg83Leu;Arg83Leu)	NA
628†	NBS	4–10	11–20	None	Myalgia in legs with normal serum free carnitine	None	c.248G>T;248G>T* (p.Arg83Leu;Arg83Leu)	NA

Null variations are in bold. Reference sequence for variants: RefSeq NM_003060.3.

*Genotype reported in Makhseed *et al* 24: Presented with axonal neuropathy at 16 months, at 3 years unresponsive to stimuli with hypoketotic hypoglycaemia. Sibling 11 years (also homozygous), no symptoms.

†Siblings.

F, female; M, male; NA, not available; NBS, newborn screening.

All patients with PCD received treatment on diagnosis. However, seven mothers were not immediately diagnosed at the time of referral because of relatively high plasma carnitine concentrations and the presence of only one variant in the *SLC22A5* gene. Genetic re-evaluation after several years revealed a second variant (c.-149G>A) in all seven cases. These individuals were not treated and follow-up data were not available (table 3; cases 127, 136, 164, 166, 167, 191, 258).

**Table 3:** Overview of patients with a likely benign form of PCD

Patient ID	Identified	Age at Dx (years)	Age initial symptoms (Years)	Age at last follow-up (Years)	Symptoms	Treatment effect	Variants DNA (Protein)	Transport activity
132	Mother	31–40	31–40	41–50	Asymptomatic*	None	c.-149G>A;−149G>A† (5’UTR;5’UTR)	20.5%
143	Mother	31–40	NA	31–40	Asymptomatic	–	c.-149G>A;−149G>A† (5’UTR;5’UTR)	27.5%
164	Mother	–	NA	21–30	Asymptomatic	–	c.-149G>A;−149G>A† (5’UTR;5’UTR)	40.0%
198	Mother	31–40	Puberty	31–40	Fatigue	None, Fishy odour	c.-149G>A;−149G>A† (5’UTR;5’UTR)	42.5%
136	Mother	–	NA	21–30	Asymptomatic	Not started	c.136C>T;−149G>A (p.Pro46Ser;5’UTR)	NA
145	Mother	31–40	41–50	41–50	Asymptomatic*	Not started	c.136C>T;−149G>A (p.Pro46Ser;5’UTR)	21.5%
156	Mother	31–40	31–40	31–40	Asymptomatic*	None	c.136C>T;−149G>A (p.Pro46Ser;5’UTR)	NA
160	Mother	31–40	NA	31–40	Asymptomatic	Not started	c.136C>T;−149G>A (p.Pro46Ser;5’UTR)	NA
166	Mother	–	31–40	41–50	Asymptomatic*	NA	c.136C>T;−149G>A (p.Pro46Ser;5’UTR)	29.5%
174	Mother	31–40	NA	31–40	Asymptomatic	–	c.136C>T;−149G>A (p.Pro46Ser;5’UTR)	NA
215	Mother	21–30	NA	21–30	Asymptomatic	Nausea	c.136C>T;−149G>A (p.Pro46Ser;5’UTR)	NA
244	Mother	31–40	NA	31–40	Asymptomatic	–	c.136C>T;−149G>A (p.Pro46Ser;5’UTR)	NA
266	Mother	31–40	Puberty	21–30	Myalgia	None, fishy odour	c.136C>T;−149G>A (p.Pro46Ser;5’UTR)	NA
551	Newborn	<1	4–10	4–10	Asymptomatic*	NA	c.136C>T;−149G>A (p.Pro46Ser;5’UTR)	27.0%
101	Mother	21–30	21–30	21–30	Asymptomatic‡	None	c.136C>T;136C>T§ (p.Pro46Ser;Pro46Ser)	29.0%
150	Mother	21–30	NA	21–30	Asymptomatic	Not started	c.136C>T;136C>T§ (p.Pro46Ser;Pro46Ser)	NA
247	Mother	21–30	Puberty	31–40	Fatigue	None, fishy odour	c.136C>T;136C>T§ (p.Pro46Ser;Pro46Ser)	NA
616	Mother	31–40	Childhood	31–40	Myalgia	None	c.136C>T;136C>T§ (p.Pro46Ser;Pro46Ser)	NA
515	Newborn	<1	1–3	4–10	Asymptomatic*	Less fatigued	c.136C>T;136C>T§ (p.Pro46Ser;Pro46Ser)	12.5%
529	Newborn	<1	NA	11–20	Asymptomatic	Fishy odour	c.136C>T;136C>T§ (p.Pro46Ser;Pro46Ser)	14.0%
561	Newborn	<1	NA	1–3	Asymptomatic	–	c.136C>T;136C>T§ (p.Pro46Ser;Pro46Ser)	NA
577	Newborn	<1	<1	1–3	Asymptomatic¶	–	c.136C>T;136C>T§ (p.Pro46Ser;Pro46Ser)	NA
632	Newborn	<1	1–3	11–20	Asymptomatic¶	–	c.136C>T;136C>T§ (p.Pro46Ser;Pro46Ser)	NA
147	Mother	21–30	NA	31–40	Asymptomatic	–	c.640_641delinsTT;−149G>A (p.Ala214Leu;5’UTR)	28.0%
191	Mother	–	NA	21–30	Asymptomatic	Not started	c.640_641delinsTT;−149G>A (p.Ala214Leu;5’UTR)	NA
202	Mother	31–40	NA	41–50	Asymptomatic	None, fishy odour	c.640_641delinsTT;−149G>A (p.Ala214Leu;5’UTR)	25.0%
120	Mother	31–40	41–50	41–50	Asymptomatic*	None	c.136C>T;695C>T** (p.Pro46Ser;Thr232Met)	10.0%
546	Newborn	<1	NA	1–3	Asymptomatic	–	c.136C>T;695C>T** (p.Pro46Ser;Thr232Met)	NA
282	Mother	31–40	31–40	31–40	Asymptomatic*	None	c.707G>A;.-149G>A (p.Cys236Tyr;5’UTR)	NA
167	Mother	–	31–40	31–40	Asymptomatic*	NA	c.1101C>G;−149G>A (p.Asn367Lys;5’UTR)	38.5%
119	Mother	31–40	Childhood	31–40	Fatigue	None, fishy odour	c.1354G>A;−149G>A (p.Glu452Lys;5’UTR)	19.5%
242	Mother	21–30	31–40	31–40	Asymptomatic*	None	c.136C>T;457G>C (p.Pro46Ser;Val153Leu)	NA
235	Mother	31–40	NA	31–40	Asymptomatic	Not started	c.1421G>A;1421G>A (p.Ser474Asn;Ser474Asn)	NA
144	Mother	31–40	11–20	31–40	Fatigue	None	c.34G>A;1340A>C (p.Gly12Ser;Tyr447Ser)	26.0%
127	Mother	–	NA	21–30	Asymptomatic	Not started	c.34G>A;−149G>A (p.Gly12Ser;5’UTR)	NA
208	Mother	21–30	NA	31–40	Asymptomatic	–	c.797C>T;797C>T (p.Pro266Leu;Pro266Leu)	18.5%
213	Mother	21–30	NA	21–30	Asymptomatic	–	c.680G>A;−149G>A (p.Arg227His;5’UTR)	NA
205	Mother	21–30	NA	31–40	Asymptomatic	None, fishy odour	c.718G>A;−149G>A (p.Ala240Thr;5’UTR)	26.0%
258	Mother	–	NA	21–30	Asymptomatic	Not started	**c.825–1G>C**;−149G>A (**Splice**;5’UTR)	NA
125	Mother	21–30	Adolescence	21–30	Fatigue	Less fatigued	**c.825–1G>C**;136C>T (**Splice**;p.Pro46Ser)	10.5%
158	Mother	21–30	NA	21–30	Asymptomatic	Not started	c.95A>G;−149G>A (p.Asn32Ser;5’UTR)	17.5%
504	Newborn	<1	NA	11–20	Asymptomatic	–	c.136C>T;**844C>T**†† (p.Pro46Ser; **Arg282Ter**)	9.5%
115	Mother	21–30	Adolescence	31–40	Fatigue	Less fatigued	c.136C>T; not identified (p.Pro46Ser)	40.5%
196	Mother	21–30	21–30	31–40	Asymptomatic‡	Reduced myalgia	c.640_641delinsTT; not identified (p.Ala214Leu)	30.5%

Null variations are in bold. Additional information on identified variants is provided in online supplemental table 1. Reference sequence for variants: RefSeq NM_003060.3. 5’UTR – variant in the 5’ untranslated region which results in an upstream translation initiation codon, as described in Ferdinandusse *et al*.^20^

*Only mentioned fatigue after specific inquiry by treating physician.

†Genotype reported in Verbeeten *et al* 33: family diagnosed through NBS; 3 adults who mentioned fatigue in hindsight.

‡Only mentioned myalgia after specific inquiry by treating physician.

§Genotype reported in Li *et al* 34: Mother diagnosed through NBS, 26 years, asymptomatic. Also reported in de Boer *et al* 35: same patient as patient 632 in the current study.

¶Initial slight motor developmental delay, corrected before 2 years of age.

**Genotype reported in Li *et al* 34: mother diagnosed through NBS, 37 years, asymptomatic but with three spontaneous miscarriages.

††Genotype reported in Sarafoglou *et al* 36: Mother diagnosed through NBS; 35 years, asymptomatic. Also reported in Schimmenti *et al* 10: mother diagnosed through NBS, 28 years, fatigue.

Class, Classification; Dx, diagnosis; Mother, mothers identified through screening of their child; NA, not available; NBS, newborn screening; PCD, primary carnitine deficiency.

### Disease severity assessment based on genetic classification

Disease was classified as severe in nine cases (clinically identified N=5, newborn N=3, mothers N=1) (table 2, online supplemental table 3), and in six cases, this classification was based on presenting symptoms, three based on the fact that patients had a genotype previously reported in a patient with severe symptoms (patients 131, 597 and 628). PCD was classified as likely benign in 44 cases (newborn n=8, mothers n=36) (table 3). For the mothers, this was based on the untreated disease course until diagnosis, for the newborns because of lack of severe disease manifestations in adult patients with the same genotype in literature or the current study. In eight newborns, the disease was classified as of unknown severity because (the combination of) variants in these patients had never been identified in patients that had reached adulthood without treatment (table 4).

10.1136/jmg-2023-109206.supp3 Supplementary data

**Table 4:** Case overview of patients with unknown disease severity

Patient ID	Identified	Age at Dx (years)	Age initial symptoms (years)	Age at last follow-up (years)	Symptoms	Variants DNA (Protein)	Transport activity
528	Newborn	<1	NA	1–3	Asymptomatic	c.136C>T;248G>T (p.Pro46Ser;p.Arg83Leu)	8.5%
554	Newborn	<1	NA	1–3	Asymptomatic	c.506G>C;1088T>C (p.Arg169Pro;Leu363Pro)	NA
603	Newborn	<1	NA	4–10	Asymptomatic	c.95A>G;136C>T (p.Asn32Ser;Pro46Ser)	NA
606	Newborn	<1	NA	1–3	Asymptomatic	c.448T>C;**760C>T** (p.Phe150Leu;**Arg254Ter**)	NA
609	Newborn	<1	NA	4–10	Asymptomatic	c.**844C>T**;−149G>A (p.**Arg282Ter**;5’UTR)	NA
627	Newborn	<1	NA	1–3	Asymptomatic	c.**844C>T;825–1G>C** (p.**Arg282Ter;Splice**)	NA
667	Newborn	<1	NA	1–3	Asymptomatic	c.610G>A;−149G>A (p.Gly204Ser;5’UTR)	NA
681	Newborn	<1	<1	1–3	Asymptomatic*	c.**760C>T**;1354G>A (p.**Arg254Ter**;Glu452Lys)	NA

Null variations are in bold. Reference sequence for variants: RefSeq NM_003060.3. Additional information on identified variants is provided in online supplemental table 1.

*Febrile convulsions with normal serum carnitine.

Dx, diagnosis; NA, not available; NBS, newborn screening.

The allele frequencies of variants identified in the different disease severity groups were in the severe patients 100% classic variants, in the likely benign group 6% classic variants and 92% screening variants and in the unknown disease severity group 56% classic variants and 44% screening variants.

**Figure 2:**
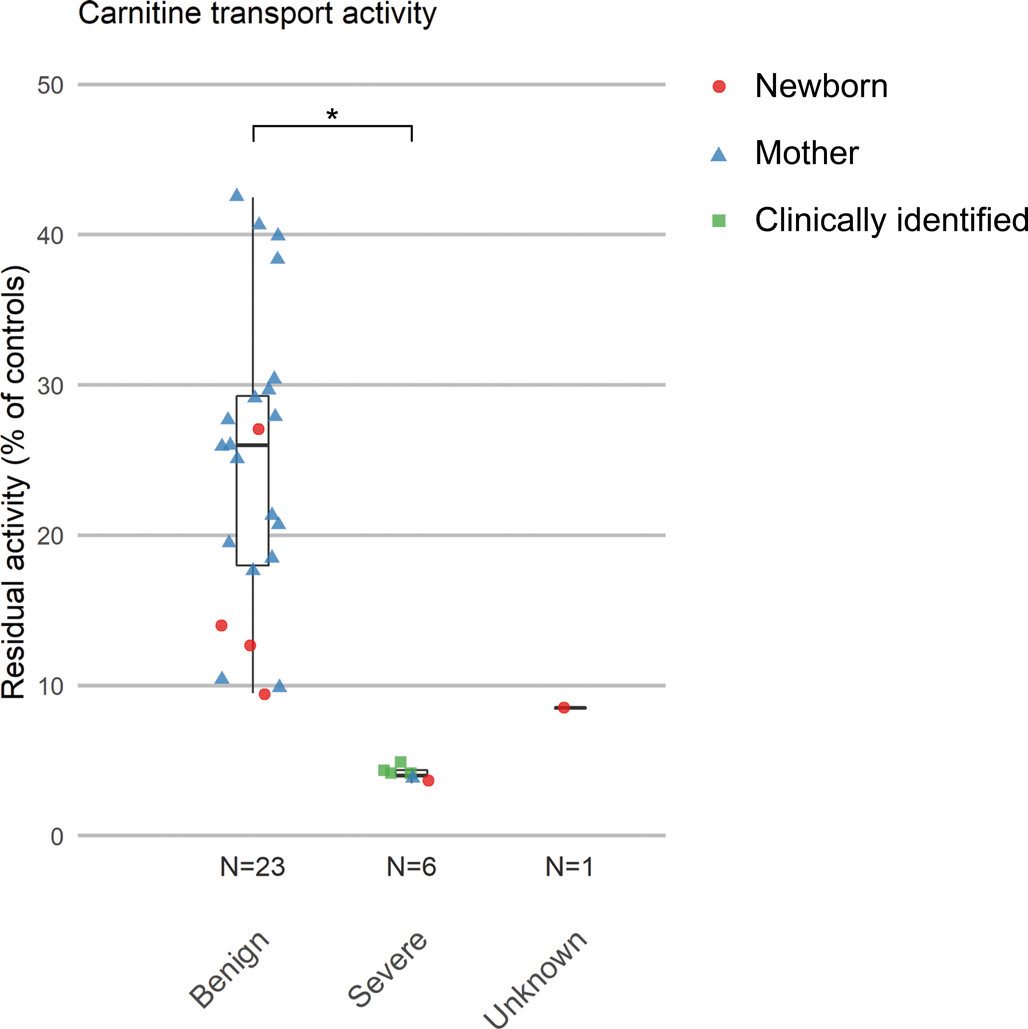
Residual carnitine transport activity of patients with PCD. The residual carnitine transport activities of patients with PCD identified by Dutch newborn screening (newborn and mothers) or clinical presentation (clinically identified) are presented. The results are grouped by disease severity based on genotypes. *P<0.001 (two-sided t-test). PCD, primary carnitine deficiency.

The median carnitine transport activity in cultured fibroblasts (median (range)) in the different groups, presented in figure 2, was as follows: in the severe group (n=6): 4.0% (range 3.5%–5.0%), in the likely benign group (n=23): 26.0% (range 9.5%–42.5%) and in the unknown disease severity group (n=1: case 528): 8.5%. The carnitine transport activity differed significantly between the severe and likely benign groups (p<0.001).

## Discussion

In this study, we evaluated 14 years of identification of PCD by the Dutch NBS programme. We demonstrate that NBS for PCD is beneficial to a small subset of identified newborns. In this study, at least 16% (n=3/19) of the newborns diagnosed with PCD can be classified as patients with severe PCD based on genetic variants. This group may be larger, as not all referred newborns were included in this study and for eight included newborns disease, severity could not be determined. One newborn with severe PCD presented with severe symptoms and recovered quickly after treatment, which was initiated promptly when results of the NBS came in (patient 502). She continues to be asymptomatic under treatment. The other two newborns with severe PCD received treatment since diagnosis (started around 2 weeks of age) and remained symptom-free, whereas an untreated patient with a similar genotype developed axonal neuropathy at 16 months and hypoketotic hypoglycaemia at 3 years of age (table 2).^24^

The benefits of treatment and follow-up are not apparent for all individuals identified with PCD by NBS. At least 78% of individuals diagnosed with PCD through NBS (newborns and mothers) are likely to remain free of severe disease symptoms into adulthood, regardless of whether they are treated or not. They are either asymptomatic or report complaints such as fatigue or myalgia, that often did not improve after treatment (table 3) and may well be due to causes other than PCD. Our results show that these individuals differ significantly from patients with severe PCD in the type of *SLC22A5* variants they carry (92% screening variants) and their residual carnitine transport activity (higher compared with the severe patients). It is important to note that screening variants include *SLC22A5* variants that are classified as class 4 or 5, based on their (expected) biochemical effect (table 3). This shows that, although the effect is expected to be pathogenic in terms of a negative effect on carnitine transporter activity, these screening variants are observed in a group of people who do not develop disease manifestations. Thus, having a pathogenic genetic variant is not the same as having a disease or being at risk for disease development.

Observations of milder disease in NBS cohorts have been made for other inborn errors of metabolism (eg, for very long-chain acyl-CoA dehydrogenase deficiency, isovaleric acidemia and β-ketothiolase deficiency). After introduction of these conditions into NBS programmes, individuals were identified with genetic variants only found through screening, in whom the identified biochemical and functional defects were milder compared with the clinically diagnosed patients. The clinical significance of the findings in these NBS patients is often unknown, because the mild phenotype might be attributable to effects of early treatment or to the milder clinical phenotype in itself. One could argue that, at least some of these individuals should not be considered patients, but individuals with a benign genetic metabolic trait.^1 2 25 26^ The present study is the first to show that this group of individuals, at least for PCD, is unlikely to suffer disease symptoms early in life if left untreated. This prompts the discussion whether all individuals identified by NBS should be treated or whether NBS should target only those with severe disease, in whom treatment prevents significant disease complications early in life. The first approach, treatment of all, ensures prevention of severe outcome, but comes at the cost of overmedicalisation. The second approach achieves effective treatment for those that may benefit, without burdening individuals with a benign metabolic trait with overmedicalisation. However, the potential of developing disease symptoms, possibly later in life in these individuals, cannot be fully ruled out. How these advantages and disadvantages are weighed is influenced by ethical, cultural and economic viewpoints. Such discussions become even more relevant as technological developments (eg, next generation sequencing) are likely to lead to further expansion of NBS programmes.^27 28^ The disadvantage of overmedicalisation should not be underestimated, as it may negatively impact quality of life of diagnosed individuals. For PCD, the most feared complications are cardiomyopathy and (ventricular) arrhythmias and this potential risk is often the reason for starting lifelong preventive carnitine treatment. This knowledge of being at risk for a potentially life-threatening arrhythmia can be especially burdensome. A study performed by Probst *et al* found that 41% of asymptomatic patients diagnosed with Brugada syndrome (a hereditary arrhythmic disease that can result in sudden cardiac death) are anxious about their health status and 24% felt a negative impact of the diagnosis on their quality of life. Younger patients were more likely to express a negative impact on their quality of life.^29^

Previously, a small number of asymptomatic adult patients with PCD were reported who presented with a sudden cardiac event.^10 30–32^ Additionally, a retrospective analysis of sudden death cases in the Faroese population revealed 13 patients with PCD, all homozygous for the c.95A>G variant. These reports in literature led to the follow-up and treatment of asymptomatic mothers detected through NBS of their child in the Netherlands and worldwide. In our study, after 14 years of follow-up of individuals referred because of low carnitine in the NBS DBS, only one of the 37 identified mothers had potentially severe PCD (based on genotype) and none had any sign of cardiac disease. Thus far, worldwide, in only two mothers identified through NBS of their child a cardiac event was reported, namely ventricular fibrillation (c.[424G>T;1463G>A];[1586+1G>T])^31^ and ventricular tachycardia (c.[95G>A];[136C>G]).^10^ Both carried one classic variant (c[424G>T;c.1463G>A] and c.[95A>G]) and one screening variant (c.[1586+1G>T] and c.[136G>C]). It remains unclear if there was a secondary cause or risk factor for ventricular arrhythmias in these two mothers. Based on the limited number of observed cardiac events in our cohort, during a period of 1279 combined life years, and in literature in NBS identified mothers, we believe this low risk of ventricular arrhythmias does not justify identification and subsequent lifelong follow-up and treatment of PCD in mothers diagnosed through NBS of their child.

In the presented cohort, disease severity remains unknown for eight newborns, since there are no natural history data available for their specific genotypes and cascade screening was not performed (table 4). Now that a novel assay for the carnitine transport activity is available,^20^ we can differentiate between a severe deficiency associated with severe clinical symptoms and a mild deficiency associated with only mild or no clinical symptoms. This assay can be used to determine the risk for newborns with unknown disease severity. Since data on transport activity of only a limited number of patients are available at this moment, this does require further substantiation. We propose the establishment of an independent international open registry for genetic, functional and phenotypic data of patients with PCD of various genetic backgrounds, to assist phenotype prediction in newly identified patients.

In conclusion, the current NBS programme in the Netherlands mainly identifies (as an incidental finding) individuals with PCD, both newborns and mothers, who are likely to remain asymptomatic. However, a small number of the newborns with PCD are likely to develop a severe outcome if left untreated and therefore undeniably benefit from identification by NBS. Based on the presence of specific genetic variants and the residual carnitine transport activity in fibroblasts, one can discriminate between individuals with a benign genetic metabolic trait and patients with severe PCD. Standardising the use of these parameters in the NBS follow-up protocol enables more specific identification of those patients with PCD who need early treatment and follow-up.

## Data Availability

Data are available on reasonable request. As this study contains identifiable data, data are available on reasonable request.
