# Supplementary material for: Newborn screening for primary carnitine deficiency: who will benefit? – a retrospective cohort study
Source: J Med Genet. 2023 Jul 24;60(12):1177–85. doi: 10.1136/jmg-2023-109206 (PMC10715524; doi:10.1136/jmg-2023-109206)
Supplement: Supplementary data [file jmg-2023-109206supp001.pdf]

Supplementary table 1. *SLC22A5* variants encountered in patients in the study cohort.

Mutation	Protein	Exon	Class.	Type	Allele freq.	First published	Other publications (homozygous)	Variant annotation
c.125T>A	p.Leu42Gln	1	3	Missense	NA	Novel		Screening variant
c.136C>T	p.Pro46Ser	1	5	Missense	4.28e-4	Schimmenti (2007)	de Boer (2013)	Screening variant
c.248G>T	p.Arg83Leu	1	5	Missense	2.68e-4	Makhseed (2004)	el Hattab (2010); Li (2010); Lin (2020)	Clinically (Makhseed 2004)
c.34G>A	p.Gly12Ser	1	4	Missense	6.95e-4	Li (2010)	Jakoby 2021	Screening variant
c.95A>G	p.Asn32Ser	1	5	Missense	2.50e-5	Lamhonwah (2002)		Clinically (Lamhonwah 2002, Rasmussen)
**c.396G>A**	**p.Trp132Ter**	2	5	Nonsense		Tang (1999)		Clinically (Tang 1999)
c.448T>C	p.Phe150Leu	2	3	Missense	NA	Novel		Screening variant
c.457G>C	p.Val153Leu	2	3	Missense	NA	Novel		Screening variant
c.506G>C	p.Arg169Pro	3	5	Missense	NA	Frigeni 2017		Screening variant
**c.597delG**	**p.Phe200Leufs**	3	5	Frameshift	NA	Yilmaz 2015		Clinically (Yilmaz 2015)
c.610G>A	p.Gly204Ser	3	3	Missense	NA	Novel		Screening variant
c.632A>G	p.Tyr211Cys	3	5	Missense	7.95e-6	Vaz (1999)	Frigeni 2017	Clinically (Vaz 1999)
c.640_641delinsTT	p.Ala214Leu	3	3	Missense	NA	Novel		Screening variant
c.646G>C	p.Val216Leu	3	3	Missense	NA	Novel		Screening variant
c.680G>A	p.Arg227His	4	5	Missense	5.96e-5	Li (2010)	Yang 2013	Screening variant
c.695C>T	p.Thr232Met	4	5	Missense	9.90e-5	Dobrowolski (2005)	Li (2010); Lin (2021)	Clinically (Li et al. 2010)
c.707G>A	p.Cys236Tyr	4	3	Missense	NA	Novel		Screening variant
c.718G>A	p.Ala240Thr	4	5	Missense	NA	Li (2010)		Screening variant
**c.760C>T**	**p.Arg254Ter**	4	5	Nonsense	1.13e-4	Tang (2002)		Clinically (Tang 2002)
c.797C>T	p.Pro266Leu	4	4	Missense	4.38e-5	Han (2014)	Chen 2013; Lin 2020	Screening variant
**c.825-1G>C**		5	5	Splice	NA	Novel		Screening variant
**c.844C>T**	**p.Arg282Ter**	5	5	Nonsense	3.98e-5	Burwinkel 1999	Wang 1999	Clinically (Burwinkel 1999)
c.934A>G	p.Ile312Val	5	3	Missense	8.63e-4	Amat di San Filippo (2008)	Li (2010)	Clinically (Li et al. 2010)
c.1088T>C	p.Leu363Pro	7	5	Missense	3.98e-6	Akpinar (2010)		Screening variant
c.1101C>G	p.Asn367Lys	7	3	Missense	NA	Novel		Screening variant
c.1232G>T	p.Gly411Val	7	5	Missense	NA	Kilic (2011)		Clinically (Kilic 2011)
c.1340A>C	p.Tyr447Ser	8	3	Missense	NA	Novel		Screening variant
c.1354G>A	p.Glu452Lys	8	5	Missense	2.78e-5	Wang (2000)	Bijarnia-Mahay (2015); Deswal (2010)	Clinically (Wang 2000)

c.1421G>A	p.Ser474Asn	8	3	Missense	3.98e-6	Novel		Screening variant
c.[424G>T;1463G>A]	p.[Ala142Ser;Arg488His]	9	5	Missense	3.19e-3	Amat di San Filippo (2006)	Frigeni 2017	Clinically (Amat di san Filippo 2006)
c.-149G>A		5'UTR	4	5UTR	NA	Ferdinandusse (2019)		Screening variant

Null-variants are in bold. Reference sequence for variants: RefSeq NM_003060.3. screening variants - variants identified in patients following screening diagnosis, and not in clinically diagnosed PCD. Clinically – variants identified in patients that have presented clinically (the reference of patients with this variant is provided). Allele freq – Allele frequencies, as provided by Gnomad (last consulted 30-5-2022).
