# Supplementary material for: Newborn screening for primary carnitine deficiency: who will benefit? – a retrospective cohort study
Source: J Med Genet. 2023 Jul 24;60(12):1177–85. doi: 10.1136/jmg-2023-109206 (PMC10715524; doi:10.1136/jmg-2023-109206)
Supplement: Supplementary data [file jmg-2023-109206supp002.pdf]

 BMJ Publishing Group Limited (BMJ) disclaims all liability and responsibility arising from any reliance placed on this supplemental material which has been supplied by the author(s)

Supplementary table 2. Characteristics of all individuals referred because of low carnitine in NBS

	NBS				Mothers referred after NBS			
	**PCD (n=19)**		**Non-PCD (n=112)**		**PCD (n=37)**		**Non-PCD (n=45)**	
**Gender** (male)	9 (47.4%)	{0}	46 (41.1%)	{0}	0 (0%)	{0}	0 (0%)	{0}
**Gestational age** (weeks)	40.1 [37.3, 41.7]	{1}	39.9 [24.4, 42.0]	{6}	-		-	
**Birth weight** (grams)	3390 [2490, 4300]	{0}	3480 [560, 4600]	{4}	-		-	
**Sibling death**	0 (0%)	{10}	1 (0.9%)	{50}	1 (3.2%)	{6}	0 (0%)	{24}
**Other IEM diagnosed after referral**	0 (0%)	{0}	0 (0%)	{0}	0 (0%)	{0}	5 (8.2%)	{0}
**Age at last follow-up** (years)	4.56 [1.03, 14.1]	{0}	0.1 [0, 8.6]	{7}	34.6 [24.8, 48.6]	{0}	31.6 [20.4, 38.3]	{3}
**Free carnitine concentration** (blood)								
First NBS Sample*	3.5 [1.8, 4.8]	{0}	4.1 [1.0, 5.0]	{5}	3.7 [1.9, 5.0]	{2}	4.1 [1.0, 5.0]	{4}
Second NBS Sample*	3.1 [1.3, 4.7]	{1}	4.3 [1.6, 5.0]	{8}	3.8 [2.0, 5.0]	{2}	4.5 [1.3, 5.0]	{6}
First sample at metabolic centre	7.5 [2.0, 12.0]	{0}	11.0 [3.1, 79.7]	{9}	6.6 [2.1, 18.8]	{3}	16.0 [2.8, 35.0]	{4}

Data presented as N (%) or median [Min-Max]. Missing data points are presented in grey, within braces. PCD - Primary carnitine deficiency; NBS - Newborn screening; IEM - Inborn error of metabolism.

* For mothers: concentration in the NBS sample of their child
