# Supplementary material for: Newborn screening for primary carnitine deficiency: who will benefit? – a retrospective cohort study
Source: J Med Genet. 2023 Jul 24;60(12):1177–85. doi: 10.1136/jmg-2023-109206 (PMC10715524; doi:10.1136/jmg-2023-109206)
Supplement: Supplementary data [file jmg-2023-109206supp003.pdf]

Supplementary table 3. Complete overview of patients with severe PCD.

Patient ID	Identified	Sex	Age initial symptoms (Years)	Age at last follow-up (Years)	Initial symptoms	Clinical course at follow-up	Treatment effect	Variants			Transport activity
								DNA	Protein	Class	
8	Clinically	M	<1	11-20	Sepsis-like presentation, Coma, Cardiomyopathy	Difficulty feeding	Complete resolution.	**c.396G>A;396G>A**	**p.Trp132Ter;Trp132Ter**	5;5	NA
10	Clinically	M	<1	31-40	Cardiomyopathy	Cardiomyopathy at 30Y	Complete resolution within 1Y. After suspending treatment for 12Y cardiomyopathy recurred with severe cardiac failure.	c.632A>G;632A>G	p.Tyr211Cys;Tyr211Cys	5;5	4%
11	Clinically	F	11-20	11-20	Ventricular fibrillation	NA	Defib. Implanted, normal ECG after 1Y.	c.1232G>T;1232G>T	p.Gly411Val; Gly411Val	5;5	5%
12	Clinically	F	1-3	21-30	Cardiomyopathy	Fatigue	Complete resolution.	**c.844C>T;844C>T**	**p.Arg282Ter;Arg282Ter**	5;5	4%
193	Clinically	F	<1	11-20	Cardiomyopathy, CMV infection, Failure to thrive	Hypoglycaemic coma with VF which lead to diagnosis at 1-3Y. Learning disability. Sudden death at 20Y	With conventional treatment (digoxine, diuretics) no improvement. Upon carnitine supplementation complete resolution.	c.632A>G;632A>G	p.Tyr211Cys;Tyr211Cys	5;5	4.5%
502	NBS	F	<1	11-20	Sepsis-like presentation	Learning disability	Fast recovery after initiating carnitine supplementation.	**c.597delG;597delG**	**p.Phe200Leufs;Phe200Leufs**	5;5	3,5%
131	Mother	F	31-40	31-40	None	Asymptomatic	None	c.248G>T;248G>T[a]	p.Arg83Leu;Arg83Leu	5;5	4%
597[b]	NBS	F	NA	4-10	-	Asymptomatic	-	c.248G>T;248G>T[a]	p.Arg83Leu;Arg83Leu	5;5	NA
628[b]	NBS	M	4-10	11-20	None	Myalgia in legs with normal serum free carnitine	None	c.248G>T;248G>T[a]	p.Arg83Leu;Arg83Leu	5;5	NA

Null variations are in bold. Reference sequence for variants: RefSeq NM_003060.3. NBS – Newborn screening; M – Male; F – Female; Y – Years; NA – Not available.
[a] Genotype reported in Makhseed et al.[24]: Presented with axonal neuropathy at 16 months, at 3Y unresponsive to stimuli with hypoketotic hypoglycemia. Sibling 11Y (also homozygous), no symptoms.
[b] Siblings
